# Thrombospondin 1 expression and angiogenesis in breast carcinoma and their relation with platelet activity

**DOI:** 10.1080/03009730902761797

**Published:** 2009-04-24

**Authors:** Cigdem Tokyol, Gulriz Ersoz, Fatma Husniye Dilek, Ercan Gencer, Mehmet Nuri Kosar, Osman Nuri Dilek

**Affiliations:** ^1^Department of Pathology, School of Medicine, Afyon Kocatepe UniversityAfyonkarahisarTurkey; ^2^Department of Physiology, School of Medicine, Ankara UniversityAnkaraTurkey; ^3^Department of General Surgery, School of Medicine, Afyon Kocatepe UniversityAfyonkarahisarTurkey

**Keywords:** Angiogenesis, breast carcinoma, platelet, platelet activity, thrombospondin 1

## Abstract

This study investigates angiogenesis and the expression of thrombospondin 1 in invasive ductal carcinoma of the breast and their possible relation to platelet counts and platelet activity. The study included 20 cases of invasive ductal carcinoma. Platelet activity was evaluated by determining thromboxane B2 and cyclic guanosine monophosphate (cGMP) levels by enzyme immunoassay (EIA).Thrombospondin (TSP) 1 and CD34 expression was studied immunohistochemically. Mean platelet count of the patient group was significantly greater than the mean platelet count of the control group (*P <* 0.05). The platelet counts were positively correlated with tumour size (*r*=0.609; *P <* 0.01). Platelet counts were higher in the patients who had grade 3 microvessel density (*P <* 0.05). The mean basal platelet cGMP level in the patient group was significantly lower than it was in the control group (*P <* 0.05). Focal TSP immunoreactivity was detectable in 5 (20%) cases in the tumour cells, and in 9 (45%) cases in the stroma. We did not find any correlation between TSP-1 staining and angiogenesis, platelet counts, platelet activity, and the histological prognostic parameters. Our study confirms the essential role of platelets in tumour growth and angiogenesis. Decreased levels of cGMP in the patient group may cause platelet hyperreactivity. Although thrombospondin 1 may be upregulated in malignant breast tissue, this is not sufficient for tumour growth and dissemination according to our results.

## Introduction

Breast cancer is the most common carcinoma in women. Invasive ductal carcinoma not otherwise specified forms the largest group of invasive breast cancers (1). It comprises between 40% and 75% in published series (2).

Breast cancer is a heterogeneous disease characterized by variable growth rates and metastatic potentials. Prognostic parameters which can be used to predict the behaviour of the tumours are sought. There are reports that observed that angiogenesis is an independent and significant prognostic marker for survival in early-stage breast carcinoma; several reports, however, have not been able to confirm the value of it (3).

Thrombospondin (TSP) is a matricellular protein that functions as an angiogenesis inhibitor (4). Studies of invasive tumours have demonstrated TSP-1 in the tumour stroma and have reported an inverse correlation between the presence of TSP-1 and tumour angiogenesis (5–8). To determine the role of TSP-1 in breast cancer and breast cancer angiogenesis, Byrne et al. have measured TSP-1 in plasma and tumour cytosols and compared levels to established clinicopathological prognostic parameters and intratumoural microvessel density. They concluded that circulating levels of TSP-1 appear to be a marker of breast cancer aggressiveness and in breast cancer may have a proangiogenic rather than antiangiogenic role (9).

Platelets are the most abundant source of TSP. It is the major protein of platelet α granules and is expressed on the cell surface upon platelet activation (10). Experimental and clinical data indicate that tumour cell–platelet interactions are important for tumour progression and metastasis (11).

In the present study, we evaluated angiogenesis and the expression of TSP-1 immunohistochemically in invasive ductal carcinoma of the breast and their possible relation to platelet counts and platelet activity in breast carcinoma patients. We have also analysed the effects of angiogenesis, TSP-1 expression, platelet counts, and platelet activation on prognostic histological parameters.

## Materials and methods

We performed a prospective clinical and laboratory study. Procedures followed were approved by the Ankara University Human Ethics Committee, and they were in accordance with the Helsinki Declaration of 1975, revised 1983.

The study group comprised 20 patients with primary operable invasive ductal carcinoma of the breast. All of the patients underwent modified radical mastectomy. The age range was 37–88 years, with a mean age of 56. They were non-smokers and were not prescribed acetylsalicylic acid in the two weeks prior to the operation.

The control group for blood sample analysis included 14 healthy women with an age range of 34–76 years, with a mean age of 50 years. They were non-smokers and were not prescribed acetylsalicylic acid in the two weeks prior to sampling. From their histories of disease, it was evident that none had ever suffered from cancer or other chronic diseases.

### Blood sample analysis

Blood samples of patients were collected prior to the operation. Platelet-rich plasma (PRP) was obtained by centrifugation of citrated blood at 300 rpm for 15 min. Platelets were separated by centrifugation at 1200 rpm for 15 min and were washed twice before being resuspended in a HEPES-buffered Tyrode solution containing 138 mM NaCl, 0.36 mM NaH_2_PO_4_, 2.9 mM KCl, 1 mM MgCl_2_, 5 mM glucose, and 20 mM N-2-Hydroxyethylpiperazine-N′-2-Ethanesulphonic Acid (HEPES) (pH 7.4). The platelet count was adjusted to 300×10^9^/L.

Platelet activation was evaluated by determining thromboxane B2 (TxB2), the stable metabolite of thromboxane A2 (TxA2), and cyclic GMP cyclic guanosine monophosphate (cGMP) levels. Thrombin (Chronolog Corp., PA, USA) in 0 8 U/ mL final concentration was added to the platelet suspension and was incubated for 15 min. The supernatant was prepared by centrifugation of thrombin-induced platelet suspension for 3 min at 10,000 rpm. TxB2 was evaluated by enzyme immunoassay (EIA) in the supernatants. The results were expressed as ng/mL (12,13).

For the determination of platelet cGMP content, PRP was incubated with sodium nitroprusside (SNP), a source of nitric oxide (NO), for 15 min, and ice-cold trisodium citrate was added. Platelets were pelleted by centrifugation, and 0.3 mL 96% ethanol was added. After evaporation of ethanol, 0.3 mL tris-ethylenediaminetetraacetic acid (EDTA) buffer was added. After 10 min, 0.1 mL of supernatant was tested by EIA (14). EIA kits were obtained from Cayman Chemical (MI, USA).

### Histopathological analysis

Surgical resected specimens from 20 patients with operable breast carcinoma were used. Tumour size was reported as the largest diameter. Tumour size was graded as: 1+(≤2 cm), or 2+(>2 cm). Tumour samples were fixed in 10% neutral buffered formalin, embedded in paraffin, cut into sections of 3 µm thickness, and stained with haematoxylin-eosin. Histological diagnosis was invasive ductal carcinoma in all cases. Tumour grading, nodal status, and vascular invasion assessment were performed on haematoxylin-eosin-stained sections. Tumour grade was determined according to the criteria of the modified version of Bloom and Richardson's method (15). Status of axillary lymph nodes was defined as negative or positive.

### Immunohistochemical studies

The section with the deepest invasion was selected and marked for the immunohistochemical analysis. The representative blocks were sectioned and mounted on poly-l-lysin-coated slides. The streptavidin-biotin-peroxidase method was performed using primary monoclonal antibodies against CD34 (c1one QBEnd/10, 1/200, Neomarkers, USA), TSP Ab-4 (clone A6.1, prediluted, Neomarkers, USA), oestrogen receptor (ER) (prediluted, Neomarkers, USA), and progesterone receptor (PR) (prediluted, Neomarkers, USA). Positive controls consisted of known positive samples of breast carcinoma for TSP Ab-4, ER, and PR. Normal identifiable vessels within the sections provided an internal control for CD34.

### Assessment of TSP-1 staining

Cytoplasmic staining in tumour cells and extracellular fibrillary staining in the stroma juxtaposed to tumour cells were defined positive. Semiquantitative analysis of the immunostaining was performed for each case. An estimate of the percentage of immunoreactive cells was determined using a score of 0–4 (0: negative; 1: 1%–25% cells stained; 2: 26%–50% cells stained; 3: 50%–75% cells stained; 4:>75% cells stained). The presence in the invasion front of a distinct TSP-positive cell zone composed of macrophages was scored into two categories: 1) No TSP-positive stromal macrophages or <10% TSP-positive cell zone composed of macrophages, 2) ≥10% TSP-positive cell zone composed of macrophages.

### Assessment of vascularity

One representative block including the invasive component of each breast tumour was selected and marked for the immunohistochemical analysis. Microvessel counts were determined by using the method of Weidner et al. (16). Microvessel density (MVD) was determined by light microscopy in areas of invasive tumour containing the highest numbers of capillaries and small venules per area at low power (40× and 100×). The area of highest neovascularization was identified. Microvessel counts were then made on a 200×field (0.15 mm^2^). Any endothelial cell or cell cluster positive for CD34 and clearly separate from an adjacent cluster was considered to be a single, countable microvessel (16). Large vessels with thick muscular walls and large vessel lumina greater than approximately eight red blood cells were excluded from the count (17). The maximum number of microvessels staining positive at 200× was graded as: 1+(<20 vessels), 2+(20–29 vessels), and 3+(≥30 vessels).

### Assessment of steroid hormone receptors

We followed Battifora and colleagues’ approach for the determination of oestrogen receptor (ER) and progesterone receptor (PR) expression and considered as positive all cases displaying easily discernible nuclear staining in more than 5% of the tumour cells (18).

### Statistical analysis

Statistical analysis of the data was performed using Mann-Whitney *U* test. The correlations between the various parameters were determined by means of the Pearson test. A *P-*value of 0.05 or less was considered significant.

## Results

### Platelet counts, platelet TxB2, and cGMP levels

**Figure 1.**
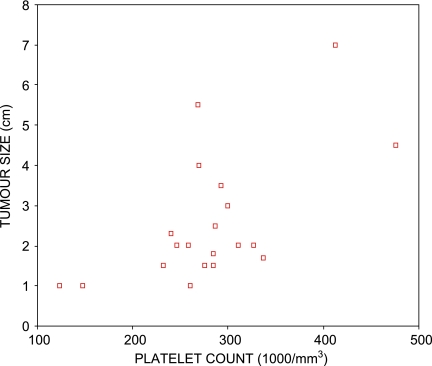
Correlation between platelet counts and tumour size.

**Figure 2.**
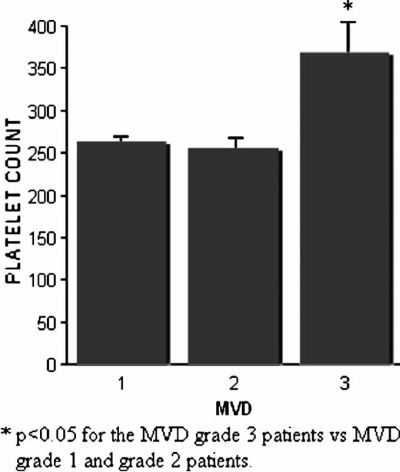
Platelet counts in the patients who had microvessel density (MVD) grade 1, grade 2, and grade 3.

There was no statistically significant difference between ages of control group and patient group. Although platelet counts were within normal ranges in both groups, the mean platelet count of the patient group was significantly greater than the mean platelet count of the control group (*P <* 0.05) (Table I). The platelet counts were positively correlated with tumour size (*r*=0.609; *P <* 0.01) (Figure 1). Platelet counts were higher in the patients who had MVD grade 3 as compared with the patients who had MVD grade 1 and MVD grade 2 (*P <* 0.05) (Figure 2). The mean basal platelet cGMP level in the patient group was significantly lower than it was in the control group (*P <* 0.05). There was no significant difference between TxB2 levels of the controls and the patients.

**Table I.** Results of the blood and tissue analysis of the groups.

	Control	Patient
Platelet count (1000/mm^3^)	236.79±12.84	281.35±17.12^a^
Platelet TxB2 (ng /mL)	76.43±10.37	84.50±6.63
Platelet cGMP (pmol /mL)	1.42±0.20	0.85±0.11^a^

Data presented as the mean±standard error of mean. ^a^ *P <* 0.05 as compared with that of control group.

### Histopathological and immunohistochemical findings

**Figure 3.**
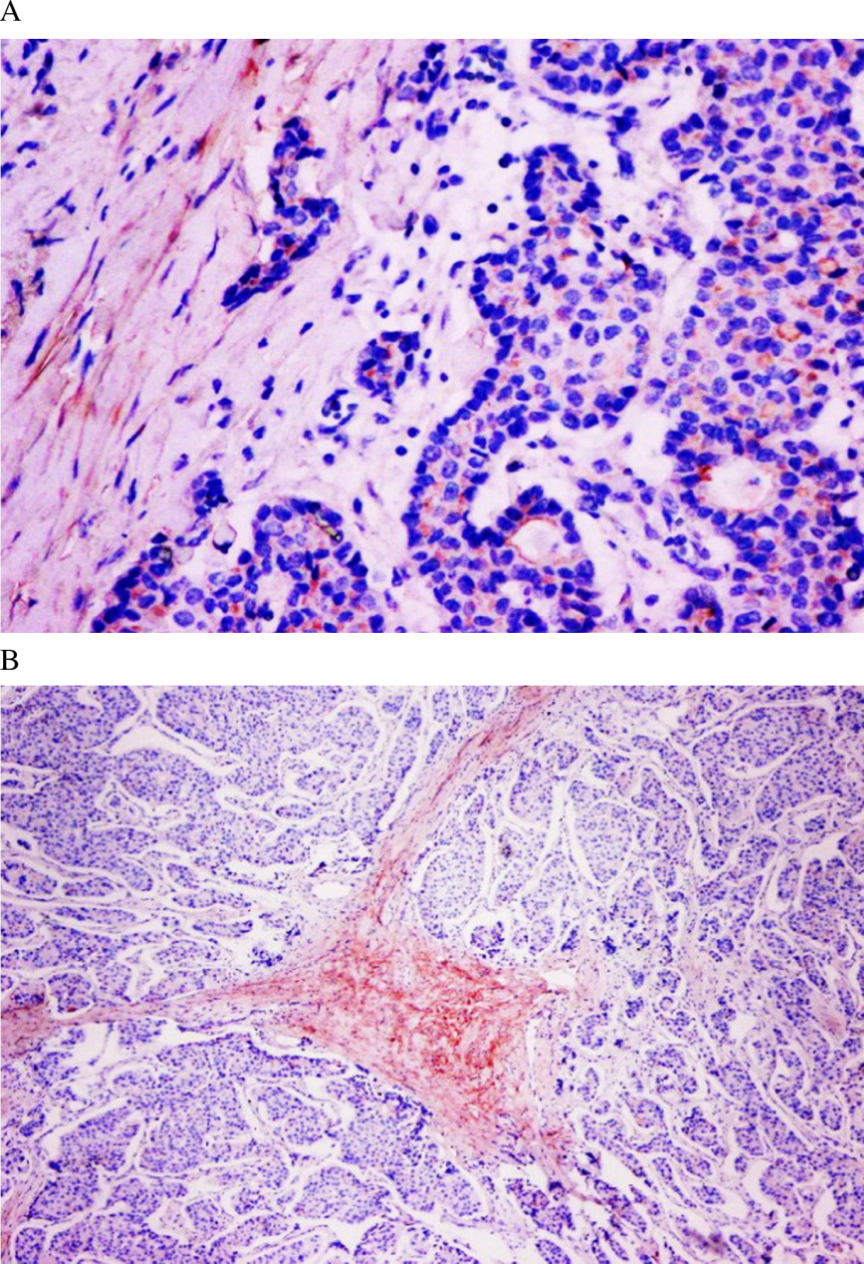
A: Cytoplasmic thrombospondin (TSP) staining with a granular texture in tumour cells and fibrillary staining in the stroma juxtaposed to tumour cells (×200). B: Fibrillary TSP staining in the tumour stroma (×40).

**Figure 4.**
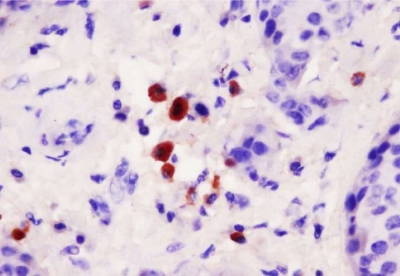
Thrombospondin (TSP)-positive macrophages adjacent to tumour cells (×400).

**Figure 5.**
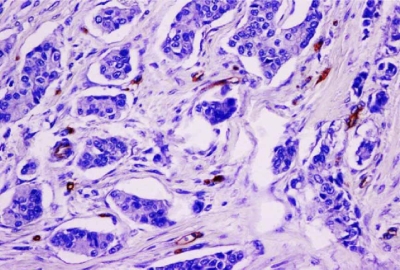
Immunohistochemical staining for endothelial cells with CD34, representative example of microvessel density (MVD) grade.

The cellular distribution of TSP in tumour cells and macrophages was cytoplasmic with a granular texture. Fibrillary staining was observed in the stroma juxtaposed to tumour cells. TSP immunoreactivity ≤25% was detectable in 5 (20%) of the cases in the tumour cells, and in 9 (45%) of the cases in the stroma (Figure 3; Table II). There was a positive correlation between tumour cell TSP staining and stromal TSP staining (*P <* 0.01). A TSP-positive cell zone composed of macrophages adjacent to tumour cells was observed in 7 (35%) of the tumours (Figure 4). No ductal or stromal staining was observed in adjacent normal breast tissue. Microvessels were noted to be most numerous at the periphery of the infiltrating tumour cells (Figure 5).

**Table II.** Histopathological and immunohistochemical findings of the 20 cases of breast carcinoma.

			MVD			Tumour Cell TSP staining		Stromal TSP staining		TSP-positive stromal macrophages	
Parameters studied	Mean age (y)	Patients (*n*)	1+	2+	3+	0	1	0	1	<10%	≥10%
Tumour size											
≤ 2 cm	60	12 (60%)	6 (30%)	4 (20%)	2 (10%)	9 (45%)	3 (15%)	5 (25%)	7 (35%)	8 (40%)	4 (20%)
> 2 cm	51	8 (40%)	4 (20%)	2 (10%)	2 (10%)	6 (30%)	2 (10%)	6 (30%)	2 (10%)	5 (25%)	3 (15%)
Histological grade ^a^											
1	55	14 (70%)	7 (35%)	5 (25%)	2 (10%)	11 (55%)	3 (15%)	9 (45%)	5 (25%)	9 (45%)	5 (25%)
2	60	6 (30%)	3 (15%)	1 (5%)	2 (10%)	4 (20%)	2 (10%)	2 (10%)	4 (20%)	4 (20%)	2 (10%)
*In situ* component											
Negative	61	11 (55%)	7 (35%)	3 (15%)	1 (5%)	8 (40%)	3 (15%)	5 (25%)	6 (30%)	7 (35%)	4 (20%)
Positive	51	9 (45%)	3 (15%)	3 (15%)	3 (15%)	7 (35%)	2 (10%)	6 (30%)	3 (15%)	6 (30%)	3 (15%)
Vascular invasion											
Negative	57	16 (80%)	8 (40%)	4 (20%)	4 (20%)	12 (60%)	4 (20%)	8 (40%)	8 (40%)	11 (55)	5 (25%)
Positive	54	4 (20%)	2 (10%)	2 (10%)		3 (15%)	1 (5%)	3 (15%)	1 v(5%)	2 (10%)	2 (10%)
Status of axillary lymph nodes											
Negative	59	8 (40%)	2 (10%)	4 (20%)	2 (10%)	6 (30%)	2 (10%)	4 (20%)	4 (20%)	5 (25%)	3 (15%)
Positive	55	12 (60%)	8 (40%)	2 (10%)	2 (10%)	9 (45%)	3 (15%)	7 (35%)	5 (25%)	8 (40%)	4 (20%)
Extranodal spread											
Negative	55	7 (35%)	5 (25%)	1 (5%)	1 (5%)	5 (25%)	2 (10%)	4 (20%)	3 (15%)	4 (20%)	3 (15%)
Positive	47	5 (25%)	3 (15%)	1 (5%)	1 (5%)	4 (20%)	1 (5%)	3 (15%)	2 (10%)	4 (20%)	1 (5%)
Status of ER											
Negative	58	9 (45%)	5 (25%)	1 (5%)	3 (15%)	7 (35%)	2 (10%)	5 (25%)	4 (20%)	7 (35%)	2 (10%)
Positive	55	11 (55%)	5 (25%)	5 (25%)	1 (5%)	8 (40%)	3 (15%)	6 (30%)	5 (25%)	6 (30%)	5 (25%)
Status of PR											
Negative	58	11 (55%)	4 (20%)	3 (15%)	4 (20%)	8 (40%)	3 (15%)	6 (30%)	5 (25%)	8 (40%)	3 (15%)
Positive	55	9 (45%)	6 (30%)	3 (15%)		7 (35%)	2 (10%)	5 (25%)	4 (20%)	5 (25%)	4 (20%)

^a^ There are no grade 3 cases.

The Pearson test did not show the existence of any significant correlations between the tumour cell TSP staining, stromal TSP staining, TSP-positive cell zone composed of macrophages, MVD, and any of the conventional tumour features, including tumour size, tumour grade, vascular invasion, status of axillary lymph nodes, extranodal spread, ER immunostatus, and PR immunostatus. There was no correlation between TSP staining and MVD.

## Discussion

Angiogenesis is the formation of new blood vessels from the endothelium of the existing vasculature and is essential for tumour growth and metastasis (19). It has been previously shown that the density of microvessels in an invasive breast carcinoma correlates with the presence of metastasis (16). There are reports that observed angiogenesis as an independent and significant prognostic marker for survival in early-stage breast carcinoma; several reports, however, have not been able to confirm the value of angiogenesis as a prognostic marker (4).

Ultrastructural studies of experimental metastasis showed that circulating tumour cells are frequently surrounded by platelets (20). Platelets may promote metastasis by releasing certain tumour growth factors and by shielding tumour cells from immune surveillance (11). It has been shown that thrombocytosis is associated with a poor prognosis in lung and endometrial carcinoma (21–23). The platelet counts were within normal ranges in the patient group, but they were significantly greater than the control group. The higher platelet counts in breast carcinoma patients and the positive correlation between platelet count and tumour diameter confirm the essential role of platelets in tumourigenesis.

Recently the hypothesis that platelets are an important factor in angiogenesis has been proposed (24,25). Platelets may affect angiogenesis by release of angiogenesis-related factors. The hypothesis is based on clinical and preclinical findings that tumour angiogenesis is dependent not only on endothelial cells and tumour cells, but also on platelet-endothelium interaction (25). We observed that platelet counts were higher in the patients who had MVD grade 3. This finding confirms the effect of platelets on angiogenesis.

TxA2 is the major vasoconstrictor and stimulator of platelet aggregation. There are inconclusive results about the role of TxA2 in tumour-induced platelet aggregation (TIPA) (26). Thrombin-induced TxA2 generation of the platelets was determined by measuring levels of TxB2, a stable metabolite of TxA2. We did not find any difference in TxB2 levels of the patients and the controls.

Intraplatelet cGMP acts to inhibit aggregation (27). It is associated with endothelial function (28). CGMP is synthesized in platelets through a soluble guanylate cyclase, activated by nitrovasodilators and NO (29). NO levels were determined in several malignant tissues (30–32). Tumour cell-associated high levels of NO were reported (30,33). Di Nicola et al. also found high levels of cGMP in malignant tissue (30). We did not evaluate NO or cGMP levels in malignant tissue, but we found lower levels of cGMP in patients. Decreased levels of cGMP may cause platelet hyperreactivity. Jurasz et al. mentioned that how the platelet-inhibitory effects of NO translate into regulation of cancer growth, invasion, and metastasis was uncertain. They thought it was an expected result because of the complexity of NO effects (26). It seems that further studies are needed to explain the role of the NO-cGMP pathway in carcinogenesis and TIPA.

TSP is a matricellular protein that does not function as structural component of extracellular matrix but interacts with matrix proteins, cell surface receptors, or other molecules that interact with the cell surface (34). Five subtypes of TSPs have been identified (35). TSP-1 was first described as a product of platelets (36). In addition to platelets, a variety of other cells have been found to contain and secrete TSP-1, including endothelial cells, smooth muscle cells, fibroblasts, pneumocytes, macrophages, monocytes, and tumour cells (37–39). TSP-1 modulates cell-cell interaction and promotes the adhesion and aggregation of platelets (40–42). It functions as an angiogenesis inhibitor (5). Using immunohistochemical techniques, Wong et al. found in 37 specimens of invasive ductal carcinoma that TSP stained strongly in the desmoplastic stroma or at the basement membrane associated with the malignant ductal epithelium. Stronger TSP staining was seen in the poorly differentiated tumours. In six cases of ductal carcinoma *in situ* TSP staining was confined to the basement membrane of the involved ducts. TSP could not be demonstrated in the basement membrane of normal breast epithelium (43).

Clezardin et al. observed a weak staining in the basement membrane of normal ductules and a strong immunostaining for TSP-1 in the basement membrane surrounding *in situ* carcinomas. In 20 cases of invasive ductal carcinomas studied, a weak staining for TSP-1 was observed in 10% of invasive cells. Excessive TSP-1 deposits were observed in desmoplasia of invasive ductal carcinomas. This finding was consistent with the fact that fibroblasts synthesize and secrete TSP-1 and that TSP-1 promotes both adhesion and growth of fibroblasts (44).

Wang-Rodriguez et al. reported that TSP-1 expression was significantly higher in malignant epithelial sources over normal and benign epithelial sources, but no difference in expression levels was evident between primary tumours with or without metastases, nor between primary and metastatic carcinomas. They observed very weak staining in normal tissues, and no TSP-1 was detected in breast tissue stromal components (45).

In the present study, only focal TSP immunoreactivity ≤20% was detectable in 5 (20%) of the tumours in the tumour cells, and in 9 (45%) of the tumours in the stroma. There was a positive correlation between tumour cell TSP staining and stromal TSP staining. Intensity of staining ranged from weak to moderate. The tumours included in our study were grade 1 and grade 2. This may be the reason for weak to moderate staining of the tumours. Stronger staining may be observed if grade 3 tumours were included. A TSP-positive cell zone composed of macrophages was observed in 7 (%35) of the tumours. Tumour-infiltrating macrophages may be a major source of TSP-1 as well as tumour cells and tumour-associated stroma. We did not find any correlation between TSP-1 staining and angiogenesis, platelet counts, platelet activity, and the histological prognostic parameters we have studied. No ductal or stromal TSP staining was observed in adjacent normal breast tissue.

The present study evaluated angiogenesis and expression of TSP-1 in invasive ductal carcinoma of the breast and their relation with platelet activity. The platelet counts were positively correlated with tumour size and were higher in the patients who had MVD grade 3. This confirms the essential role of platelets in tumour growth and angiogenesis. We found increased platelet counts and decreased cGMP levels in the patient group. Since cGMP level is accepted as an index of NO production, we thought that further studies are needed to explain the role NO-cGMP pathway in carcinogenesis and TIPA. Although TSP-1 may be upregulated in malignant breast tissue, this is not sufficient for tumour growth and dissemination according to our results.
